# Identification and characterization of satellite DNAs in two-toed sloths of the genus *Choloepus* (Megalonychidae, Xenarthra)

**DOI:** 10.1038/s41598-020-76199-8

**Published:** 2020-11-05

**Authors:** Radarane Santos Sena, Pedro Heringer, Mirela Pelizaro Valeri, Valéria Socorro Pereira, Gustavo C. S. Kuhn, Marta Svartman

**Affiliations:** 1 Laboratório de Citogenômica Evolutiva, Departamento de Genética, Ecologia e Evolução, Instituto de Ciências Biológicas, Universidade Federal de Minas Gerais, Belo Horizonte, MG Brazil; 2 Fundação de Parques Municipais e Zoobotânica, Belo Horizonte, MG Brazil

**Keywords:** Cytogenetics, Evolutionary biology, Genome, Molecular evolution, Phylogenetics

## Abstract

*Choloepus*, the only extant genus of the Megalonychidae family, is composed of two living species of two-toed sloths: *Choloepus didactylus* and *C. hoffmanni*. In this work, we identified and characterized the main satellite DNAs (satDNAs) in the sequenced genomes of these two species. SATCHO1, the most abundant satDNA in both species, is composed of 117 bp tandem repeat sequences. The second most abundant satDNA, SATCHO2, is composed of ~ 2292 bp tandem repeats. Fluorescence in situ hybridization in *C. hoffmanni* revealed that both satDNAs are located in the centromeric regions of all chromosomes, except the X. In fact, these satDNAs present some centromeric characteristics in their sequences, such as dyad symmetries predicted to form secondary structures. PCR experiments indicated the presence of SATCHO1 sequences in two other Xenarthra species: the tree-toed sloth *Bradypus variegatus* and the anteater *Myrmecophaga tridactyla*. Nevertheless, SATCHO1 is present as large tandem arrays only in *Choloepus* species, thus likely representing a satDNA exclusively in this genus. Our results reveal interesting features of the satDNA landscape in *Choloepus* species with the potential to aid future phylogenetic studies in Xenarthra and mammalian genomes in general.

## Introduction

A significant part of eukaryotic genomes, ~ 30% in some plants to more than 50% in some insects and mammals, is composed of tandemly organized highly repetitive sequences, known as satellite DNAs (satDNAs) (reviewed in Ref.^1^). In general, satDNAs differ from other tandemly repetitive sequences by their organization, which consists of long arrays that can extend up to megabases in length. SatDNAs are major components of the constitutive heterochromatin present in fundamental chromosome structures, such as centromeres and telomeres (reviewed in Refs.^1,2^).

They also have been shown to be important components of chromosome organization, pairing, and segregation. For instance, their transcripts have been reported to participate in centromeric activity and genomic regulation^3–5^. Some satDNAs also have protein binding motifs such as the CENP-B motif which, together with the CENP-B protein, is known to be involved in kinetochore structuring by helping the assembly of the CENP-A protein in mammals^6–8^. Both the CENP-B protein and the CENP-B box motif are largely conserved in mammalian centromeres, but despite this broad conservation, the role of the CENP-B proteins is still poorly understood (reviewed in Ref.^8^).

Moreover, around 50% of some studied satDNAs have short inverted repeat (short dyad symmetry) sequences within their monomers, which have been reported as essential to chromatin structure and/or function^4,7,9–11^. Short dyad symmetry sequences have been identified in satellite DNA-free centromeres and in centromeric satDNAs which lack CENP-B boxes^7^. Those dyad symmetries are predicted to adopt non-B-form DNA structures such as cruciform, hairpins, triplexes, and single-stranded DNA, which are commonly identified in functional centromeres^4,7^.

It is important to note that functional centromere sequences (those associated with CENP-A) are restricted to relatively short segments of DNA nested within megabase arrays of pericentromeric satDNAs, each of them having different epigenetic compositions^1,11^. Although pericentromeric satDNAs are involved in centromere maintenance and stability, the factors determining their boundaries and intrinsic differences with functional centromeric sequences are not fully known^1^.

SatDNAs are important components in the evolution of eukaryotic genomes. They can evolve three times faster than intergenic regions, which often results in significant differences between sequences, even among closely related species (reviewed in Ref.^1^). This rapid evolution is thought to be a consequence of mechanisms such as unequal crossing-over, gene conversion and replication slippage^12^, which are all related with the process known as molecular drive, described by Dover^13^. Because new mutations are constantly spread by molecular drive, intraspecific satDNA arrays are often composed of very similar tandemly repeated sequences that have the potential to be used as species-specific markers.

The study of repetitive DNAs has been significantly advanced with the introduction of next-generation sequencing technologies and high-throughput in silico analyses of genomes (reviewed in Ref.^1^). One of the tools used in these studies is RepeatExplorer, a pipeline that identifies repetitive DNA sequences de novo in genomes, using the raw reads without the need of a reference library of known repetitive sequences^14^. This pipeline performs graph-based clustering analyses, identifying read similarities by comparing pairwise reads all-to-all, before grouping them into clusters.

Xenarthra is a basal eutherian group which originated and diversified entirely in South America^15,16^. With 31 recognized extant species, this superorder is divided into two orders: Cingulata, represented by armadillos; and Pilosa, composed by anteaters (Vermilingua) and sloths (Folivora)^17^. Despite its importance as a basal placental group, Xenarthra has been poorly studied in comparison with other mammals, mostly because of their strict geographic distribution and collection difficulty because of their natural behavior. Hence, more information about their ecology and genetics is essential to a better characterization of the group^16^.

Studies on the repetitive DNA fraction of Xenarthra genomes have been mostly restricted to the identification of retrotransposon families. For instance, LINE (Long Interspersed Element) and SINE (Small Interspersed Element) families have been described in six species: the sloths *Choloepus hoffmanni* and *Bradypus tridactylus*^18,19^, the anteaters *Tamandua tetradactyla* and *Myrmecophaga tridactyla*^18,20^, and the armadillos *Dasypus novemcinctus* and *Euphractus sexcinctus*^18,21^. Currently, the only Xenarthra species with an identified satDNA sequence is the armadillo *D. novemcinctus*^22^, which has a satDNA with ~ 173 bp monomers. Mapping by fluorescence in situ hybridization (FISH) revealed that this satDNA was present in the centromeres of all chromosomes in this species^22^.

Two-toed sloths are the only extant representatives of the Megalonychidae family, composed by the single living genus *Choloepus*^23^, with two species: *C. didactylus* and *C. hoffmanni*. Both species inhabit the tropical forests of South and Central America with a small overlap area of occurrence in the Amazon forest in Peru, southwestern Amazonas state and Acre state in Brazil. These two species can be differentiated mainly by morphological characters, such as pelage color^24^, osteological features^25^, the mitochondrial *COI* and *Cyt-b* genes, and the nuclear gene *Enamelin*^26,27^. Cytogenetic analyses of *Choloepus* have been mostly based on simple karyotypic descriptions without banding patterns^26,28–34^. These studies revealed a complex and confusing karyotypic scenario with significant variation in diploid numbers in *C. didactylus* (2n = 52–67) and less variation in *C. hoffmanni* (2n = 49–53), with translocations between the Y chromosome and different autosomes, occurrence of X0 females, and unpaired chromosomes described as B chromosomes.

In this work we identified and characterized the most abundant satDNA sequences from the *C. didactylus* and *C. hoffmanni* genomes using in silico methods. In addition, we mapped these sequences in the chromosomes of *C. hoffmanni*. This is the first study to identify, characterize and map satDNAs in sloths, revealing interesting aspects of the centromeric and repetitive fraction of their genomes.

## Results

### In silico identification and analysis of satDNAs

The RepeatExplorer2 analysis identified two abundant putative satDNAs in the *C. didactylus* and *C. hoffmanni* genomes, which we named SATCHO1 and SATCHO2 (Supplementary data 1 and 2) (Table 1). The analysis indicated differences in the proportion of satDNAs in the two species: the satDNA content represents > 13% of the *C. didactylus* genome, whereas this value is approximately 3% in *C. hoffmanni*.

**Table 1:** Putative satDNAs identified by RepeatExplorer2 in *C. didactylus* and *C. hoffmanni*.

Satellite DNA	*C. didactylus*		*C. hoffmanni*	
	SATCHO1	SATCHO2	SATCHO1	SATCHO2
Satellite confidence	High	Low	High	Low
Satellite probability	0.986	0.0425	0.992	0.0426
Consensus size	117 bp	2292 bp	117 bp	2292 bp
Genome proportion	13%	0.62%	2.6%	0.23%
AT content	58.97%	54.97%	58.97%	54.97%

SATCHO1, the most abundant satDNA sequence in both species, has ~ 117 bp monomers, low levels of inter-repeat nucleotide variability (~ 3% on average) and AT content of ~ 59%. This satDNA represents 13% of the *C. didactylus* and 2.6% of the *C. hoffmanni* genomes. SATCHO2 is the second most abundant satDNA and has ~ 2292 bp monomers, inter-repeat nucleotide variability of ~ 24% on average and AT content of ~ 55%. It corresponds to 0.62% and 0.23% of the *C. didactylus* and *C. hoffmanni* genomes, respectively. Although SATCHO1 and SATCHO2 sequences are abundant in both genomes, we did not identify similar sequences in any other species on Repbase or in searches against all sequences from the non-redundant nucleotide collection in Genbank (accessed in 03/01/2020).

**Figure 1:**
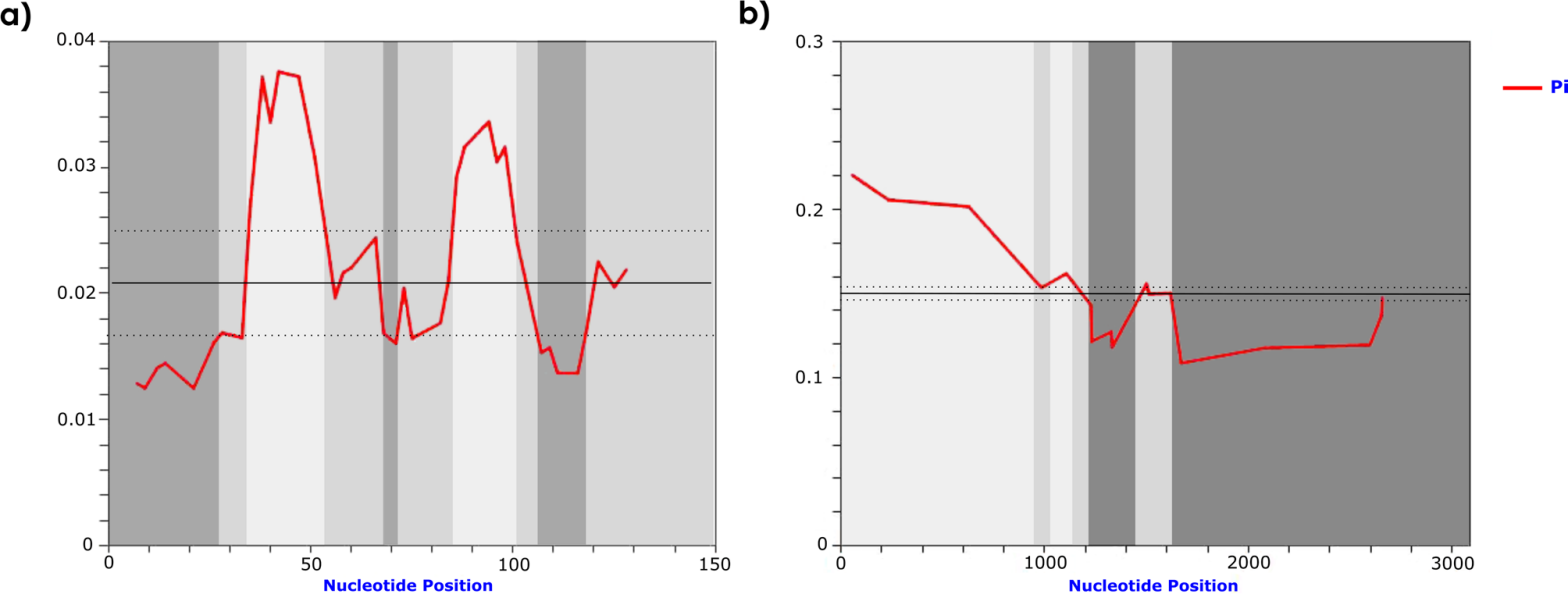
Identification of conserved (dark grey) and variable (light grey) satDNA segments of *C. didactylus* and *C. hoffmanni* by sliding window analysis. Sliding window of 10 bp for (**a**) SATCHO1 and (**b**) SATCHO2. Nucleotide diversity (Pi) is indicated by the red line, average nucleotide diversity is indicated by the solid line, and average diversity ± 2 SD is indicated by the dotted line.

The analysis of nucleotide variability along both satDNAs revealed the presence of conserved regions within their monomers (Fig. 1), even though satDNAs are expected to evolve neutrally, revealing regions under potential selective constraints.

### Phylogenetic and NMDS analyses

**Figure 2:**
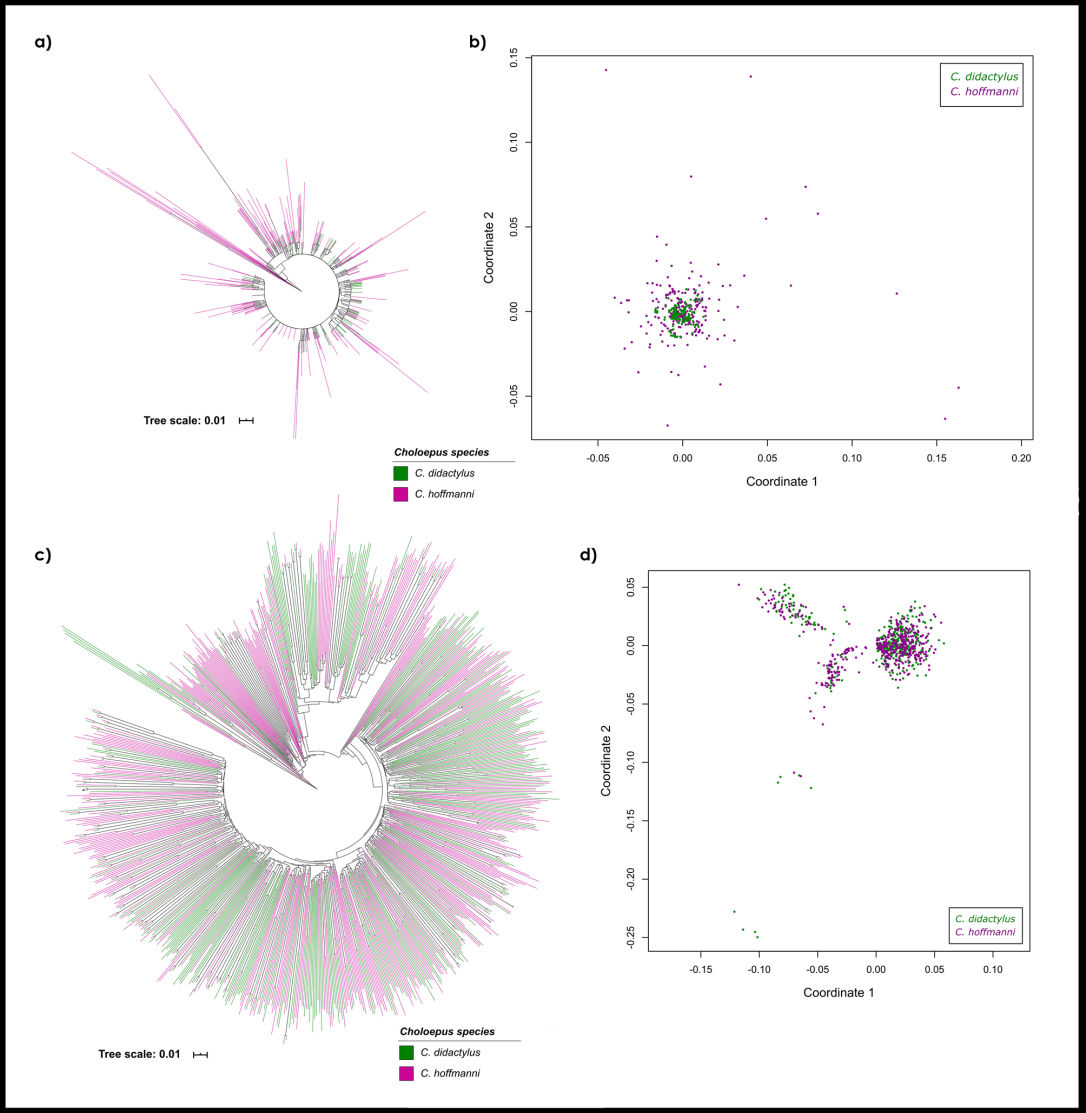
Comparative phylogenetic analyses of (**a**) SATCHO1 and (**c**) SATCHO2 sequences between *C. didactylus* and *C. hoffmanni* inferred by the Neighbor-Joining method with 1000 bootstraps. Minimum bootstrap support is 50%. Non-metric Multidimensional Scaling (NMDS) of evolutionary divergence among (**b**) SATCHO1 and (**d**) SATCHO2 sequences between *C. didactylus* and *C. hoffmanni*. The ordinations in (**b**) and (**d**) represent Euclidian distances for four dimensions. Each color represents sequences from one *Choloepus* species: *C. didactylus* (green) and *C. hoffmanni* (magenta).

In order to infer the interspecific similarity between copies of SATCHO1 and SATCHO2 in *C. didactylus* and *C. hoffmanni*, we constructed a Neighbor-Joining tree using a sample of copies from each satDNA. The resulting tree showed that satDNA copies from both species are very similar and did not segregate into species-specific branches for SATCHO1 and SATCHO2 sequences (Fig. 2a,c).

We also estimated the pairwise distance values of the same set of sequences to generate NMDS ordinations for their Euclidean distances. The results also did not reveal any clear topological segregation between copies from each species (Fig. 2b,d). Nevertheless, each satDNA appeared to evolve under distinct evolutionary rates, as evidenced by their heterogeneous distribution across the NMDS ordinations.

Overall, both analyses indicate that the satDNA sequences from *C. didactylus* and *C. hoffmanni* have not diverged enough to segregate into species-specific clusters.

### Chromosome mapping of SATCHO1 and SATCHO2

**Figure 3:**
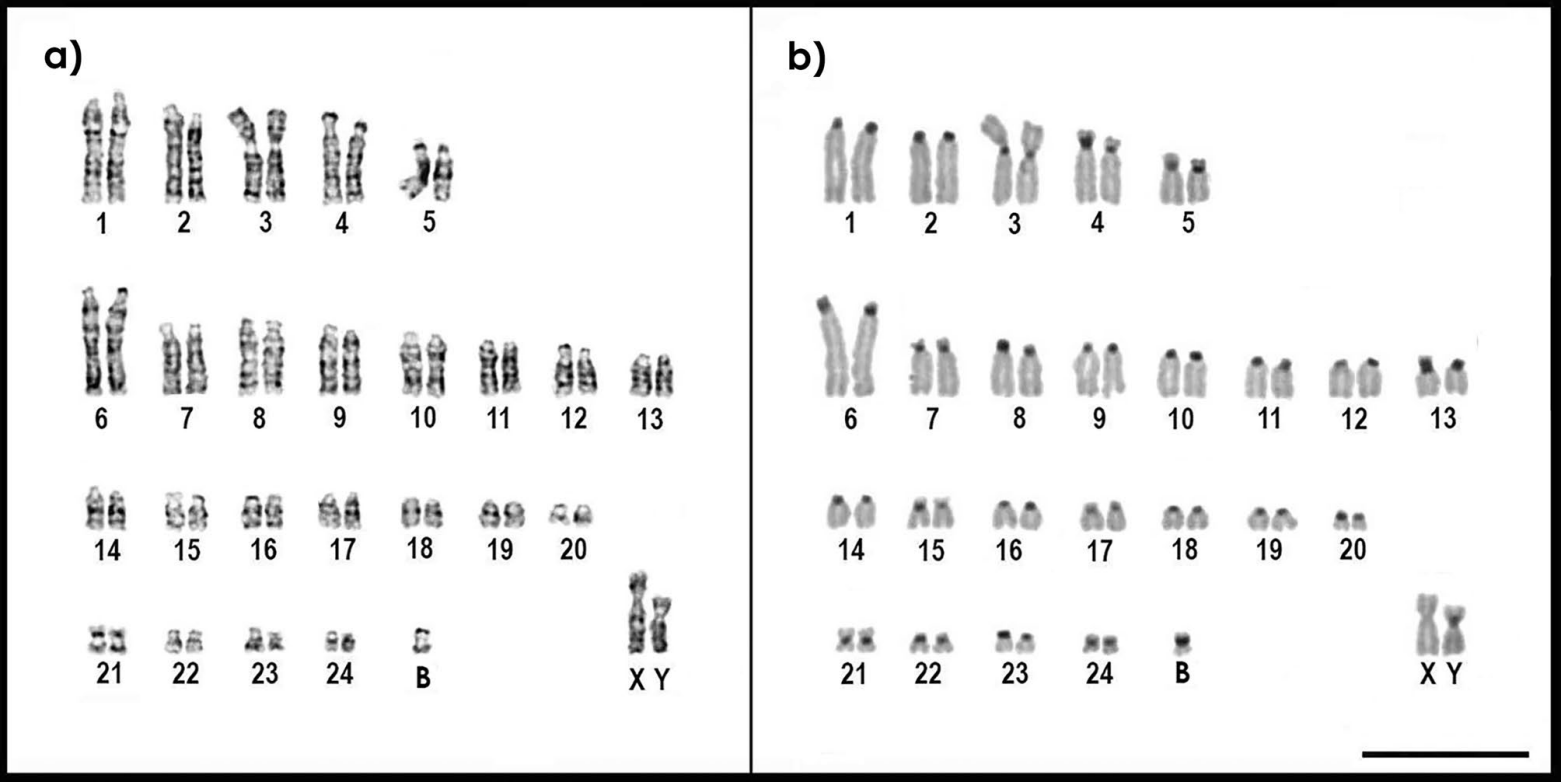
Karyotype of *Choloepus hoffmanni* (2n = 51) after: (**a**) GTG-banding and (**b**) CBG-banding. Bar = 10 µm.

The *C. hoffmanni* individual we studied presented a karyotype with a diploid number 2n = 51. GTG-banding allowed the identification of all chromosome pairs and of an odd chromosome, which we identified as a B chromosome (Fig. 3a). The CBG-banding revealed the presence of constitutive heterochromatin in the centromeric regions of all chromosomes, except the X (Fig. 3b).

Our specimen has the same karyotype described earlier by Svartman et al.^34^ for *C. hoffmanni* (2n = 50), from which it differs by the presence of the extra odd chromosome and by an inversion in pair 3 (metacentric in our specimen and acrocentric in the one previously described).

**Figure 4:**
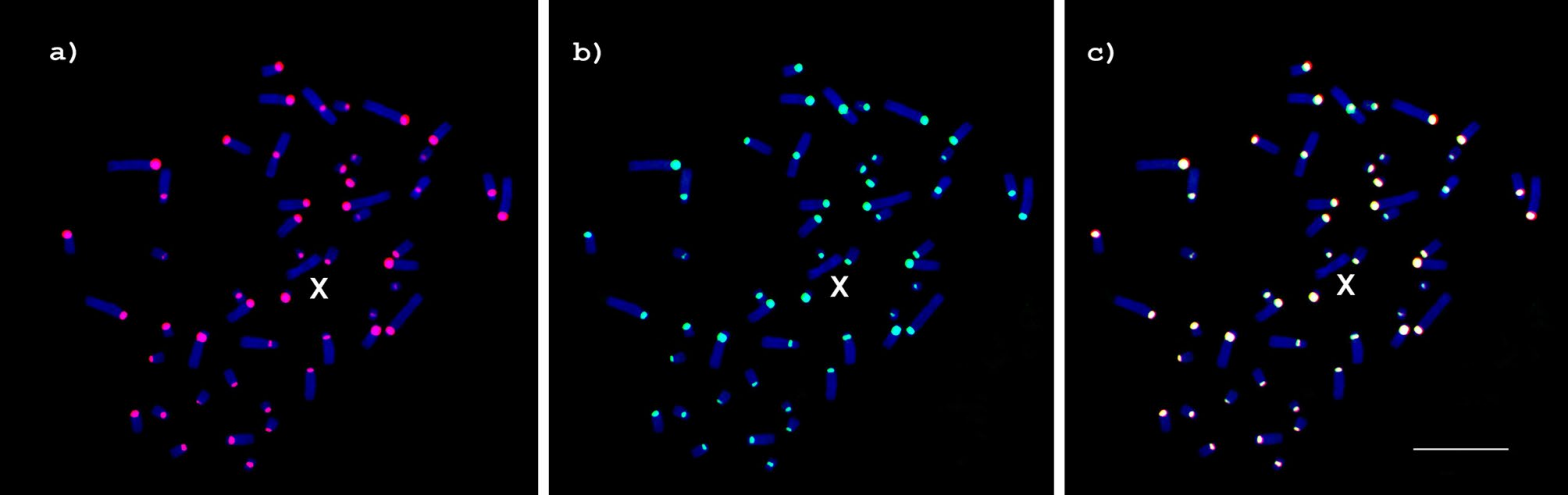
Metaphases of *Choloepus hoffmanni* after FISH using (**a**) SATCHO1 (red) and (**b**) SATCHO2 (green) as probes. (**c**) Merged signals from (**a**) and (**b**). Chromosomes were counterstained with DAPI. Note the signals in the centromeric regions of all chromosomes, except the X. Bar = 10 µm.

SATCHO1 and SATCHO2 were both FISH mapped in the centromeric regions of all *C. hoffmanni* chromosomes, except the X (Fig. 4), coinciding with the constitutive heterochromatin revealed after CBG-banding (Fig. 3b). This finding suggests that both satDNAs could play a functional role in the centromeres of *C. hoffmanni*.

### Centromeric features of SATCHO1 and SATCHO2

**Figure 5:**
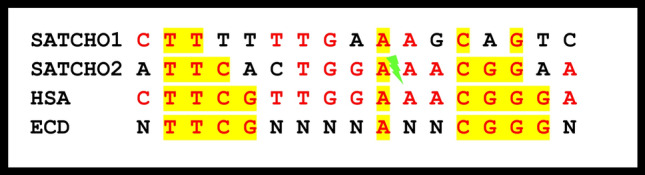
CENP-B motifs identified in sequences from SATCHO1 and SATCHO2 aligned with *Homo sapiens* (HSA) and the evolutionary conserved domain (ECD). In red: conserved bases compared with HSA. In yellow: conserved bases compared with ECD. The CENP-B motif found in SATCHO2 is composed of two different fragments separated by ~ 140 bp, as indicated by the green symbol.

Because SATCHO1 and SATCHO2 were located in the centromeric regions of *C. hoffmanni* chromosomes, we searched for putative CENP-B box-like motifs within these satDNA sequences. These motifs are typical of mammalian centromeric sequences and are thought to associate with kinetochore proteins^6,35,36^. We found that SATCHO1 has a motif with 5 of the 9 conserved nucleotides present in the evolutionary conserved domain (ECD) box (TTCGNNNNANNCGGG)^22,37^, having 73% of overall similarity with its canonical structure and sharing 59% sequence similarity with the human CENP-B box (Fig. 5). Interestingly, this putative CENP-B box-like motif from SATCHO1 overlaps with the conserved region identified by DnaSP analysis on its distal portion (Fig. 1a). In the SATCHO2 sequence, we identified two segments separated by ~ 140 bp which together form a putative CENP-B box-like motif (Fig. 5). These segments however constitute a broken motif and are thus unlikely to compose a functional sequence.

**Figure 6:**
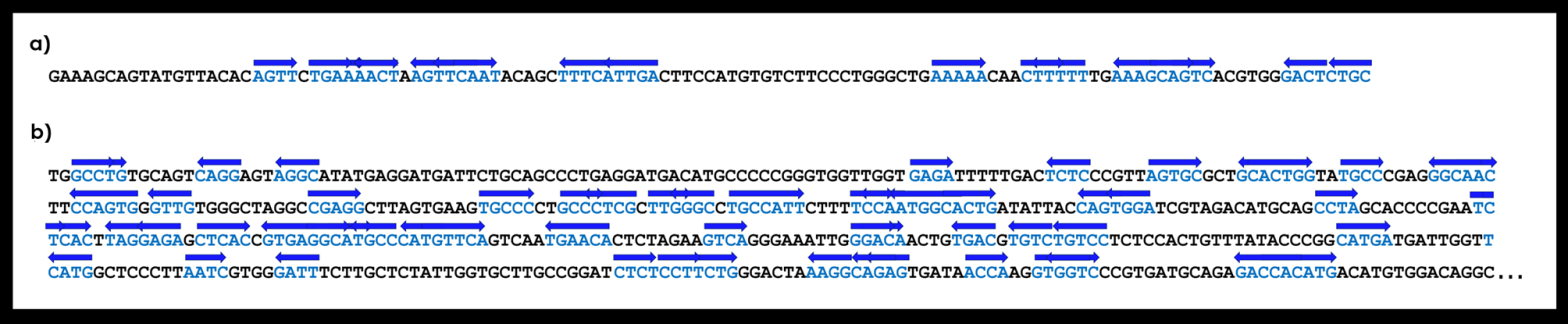
(**a**) SATCHO1 and (**b**) SATCHO2 sequences with dyad palindrome sequences. Each palindrome pair is represented by the blue color and the direction arrow above them.

**Figure 7:**
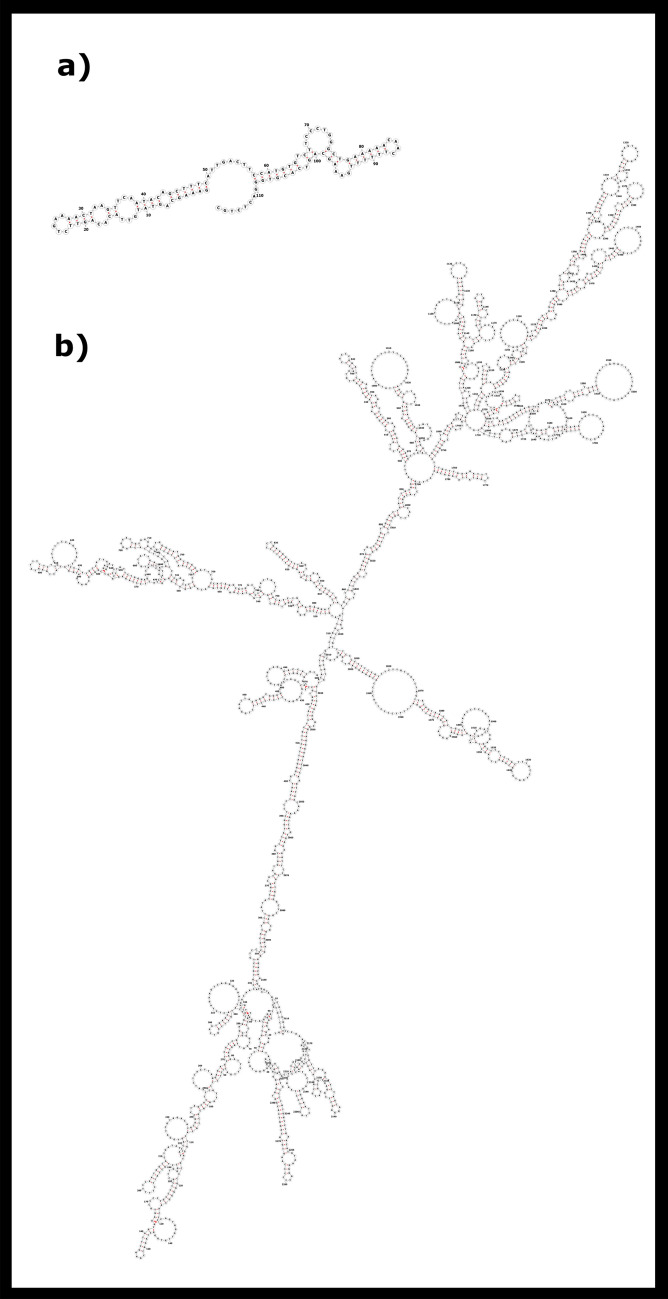
The optimal secondary structure of (**a**) SATCHO1 and (**b**) SATCHO2 predicted by RNAfold.

We also found some small palindromic sequences with 4–5 bp on both satDNAs (Fig. 6). As we have mentioned, these dyad symmetries have the potential to form secondary DNA structures which are commonly found on functional centromeric sequences. Indeed, the analysis of nucleic acid folding prediction showed that several segments within SATCHO1 and SATCHO2 have the potential to form stable DNA secondary structures (Fig. 7). These results indicate that both satDNAs contain structural hallmarks of functional centromeric sequences.

### SATCHO1 and SATCHO2 in other Xenarthra

In order to verify if the satDNAs identified in *Choloepus* are also present outside the genus, we conducted Blastn searches against assembled Xenarthra genomes. For SATCHO1 we got hits in multiple contigs of the assembled genomes of *B. variegatus*, *M. tridactyla* and *T. tetradactyla*. However, the maximum number of tandemly repeated copies retrieved in a single contig was 42 on *B. variegatus*, and 3 on *M. tridactyla* and *T. tetradactyla*. In contrast, searches on both *Choloepus* species returned hundreds of contigs including considerable results, with some of them having up to 295 tandemly repeated copies of SATCHO1.

Blastn searches on different assembled Xenarthra genomes using SATCHO2 as a query returned hits in multiple contigs only in *B. variegatus.* However, we only found up to three tandemly repeated copies in this species. In the genus *Choloepus* however, Blastn searches retrieved hundreds of contigs with hits, and up to 60 tandemly arranged copies in a single contig. Interestingly, although SATCHO1 and SATCHO2 have a centromeric localization, we did not find contigs including both satDNA sequences in none of our Blast searches.

We also performed PCR experiments using the SATCHO1 and SATCHO2 primers in the genomic DNAs of the three-toed sloth *B. variegatus* and the giant anteater *M. tridactyla*. SATCHO1 homologous sequences were amplified from both species (Supplementary Fig. 1), which was confirmed by cloning and sequencing. The two sequenced copies from *B. variegatus* showed an average of ~ 2% nucleotide divergence from the *Choloepus* SATCHO1 consensus sequence*,* whereas the two copies of *M. tridactyla* presented an average of ~ 1% nucleotide divergence. FISH with the SATCHO1 probe in *M. tridactyla* chromosomes did not produce any signal (data not shown). The SATCHO2 sequence did not amplify by PCR with the genomic DNAs of neither *B. variegatus* nor *M. tridactyla* (Supplementary Fig. 2).

These results suggest that, although SATCHO1 and SATCHO2 are present outside the genus *Choloepus*, these sequences are not distributed as abundant long arrays of tandem repeats in other Xenarthra genera, in which they should not be classified as satDNAs.

## Discussion

In this work we identified two novel centromeric satDNAs in the genomes of *C. didactylus* and *C. hoffmanni*, which could potentially have a centromeric function. Although both species have the same satDNAs, the results from RepeatExplorer revealed some marked differences in the genome proportion of these sequences in each species (Table 1). It is important to note that both species have approximately the same genome size (~ 3.3 Gb) as indicated by their sequencing projects (*C. didactylus* accession: GCA_004027855.1, *C. hoffmanni* accession: GCA_000164785.2). Despite the possibility that these observed differences reflect a real interspecific variation, it is also likely that they constitute artifacts derived from distinct values of genome coverage and/or sequencing platforms used for each species (Illumina HiSeq 2000 for *C. hoffmanni*, and Illumina HiSeq 2500 for *C. didactylus*). Although it is currently not possible to rule out any of these possibilities, the high sequence similarity and comparable number of Blastn results in both satDNAs between species indicate that a real large difference in abundance is unlikely. Indeed, a recent study demonstrated that different sequencing platforms, or even different versions of the same platform, have their own biases in representing the true proportion of highly abundant repeats^38^.

Our phylogenetic and NMDS results revealed that both satDNAs do not segregate into different branches in a species-specific manner. That result was unexpected, considering that satDNAs usually evolve rapidly through the process of molecular drive, which also tends to produce a high degree of intra-species sequence homogeneity^13^. Hence, this high level of sequence identity could be explained by one or more of the following hypotheses: (i) *C. didactylus* and *C. hoffmanni* share a very recent common ancestor; (ii) they display a slow rate of molecular evolution; (iii) they went through a recent process of hybridization; (iv) or that these satDNAs sequences have been conserved by selective pressures. Regarding the first possibility, previous molecular data from different studies showed considerable variation in the estimated divergence between *C. didactylus* and *C. hoffmanni*. For instance, using the mitochondrial gene *Cyt-b*, the split of the two *Choloepus* species was estimated at ~ 18.7 Mya with Bayesian inference and ~ 5.8 Mya with Median Joining Network^27^. Gibb et al.^39^ estimated the split varying from 3.5 to 16.7 Mya, based on mitogenomic shotgun data with Bayesian and maximum likelihood phylogenetic inferences. Hence, these estimates of divergence times argue against the hypothesis of a very recent common ancestor of *C. didactylus* and *C. hoffmanni*. In relation to the second hypothesis, *Choloepus* species have been shown to display a relatively slow rate of molecular evolution when compared to other Xenarthra groups^39^, although the reason for that is not fully understood. However, even considering that a slower rate of molecular evolution could partially explain the high sequence identity found between these satDNAs, it does not seem likely that sequences evolving neutrally would keep this level of conservation after several million years. As to the third possibility, it is worth mentioning that the two *Choloepus* species inhabit some overlapping areas of the Amazon forest and there is no precise information about the collecting areas of most specimens studied^27^. Hence, the chance of interspecific hybridization cannot be ruled out. Finally, the hypothesis that SATCHO1 and SATCHO2 could have been conserved by selective pressures is currently more difficult to evaluate beyond the evidence we provided for a putative centromeric function, as its likelihood also depends on the exclusion of the first three possibilities. Nevertheless, considering all the evidence provided here and elsewhere, we suggest that the sequence conservation of these satDNAs between *C. didactylus* and *C. hoffmanni* likely derive from a combination of selective pressures and a slow rate of molecular evolution.

More importantly, our results revealed that both satDNAS are located in the centromeric regions of all *C*. *hoffmanni* chromosomes, except the X (Fig. 4a). It has been suggested that the most abundant tandem repeat in a given genome likely corresponds to its centromeric sequence^40^, a feature that was observed for SATCHO1 in *C. hoffmanni*, and presumably also in *C. didactylus*. Although *C. hoffmanni* had the two satDNAs mapped to centromeric regions, the resolution of our results does not enable us to determine how they are distributed along the centromeric heterochromatin and if this distribution varies among chromosomes. As we have mentioned, it is also not possible to determine if one or both satDNAs are part of the functional centromere. Further analyses using long sequencing reads, chip-seq with CENP-A antibodies and immuno-fiber FISH experiments would be important to address these issues.

In addition, we found conserved regions in SATCHO1 and SATCHO2, which include motifs sharing similarities with CENP-B box-like sequences (Fig. 5). Although the CENP-B box-like motif of SATCHO2 is disrupted by an intruding sequence, and thus is probably non-functional, its presence indicates that this large satDNA might have been previously involved in centromeric activity during the evolution of *Choloepus*. It is also possible that SATCHO2 currently has a secondary centromeric function, unrelated with the activity carried out by satDNAs containing CENP-B box-like sequences. In any case, the conservation of such regions in these satDNAs suggests that they could be under some sort of selective constraint. The fact that SATCHO1 and SATCHO2 also have an enrichment of symmetric sequences capable of forming non-B DNA forms and secondary structures argues for their putative centromeric function, as these nucleotide arrangements are thought to interact with centromere components^7,11^.

Taken together, our data suggest a putative functional role for these satDNAs, which would explain their centromeric localization in *C. hoffmanni* and remarkable conservation in both *Choloepus* species. Similar results were reported in rodents of the genus *Peromyscus*, in which the centromeric satDNA PMsat was found in the centromeres of seven species^41^. Similarly to our results, PMsat monomers presented small sequence variation and shared similarities with the human CENP-B box-like motif. Based on these observations, the authors suggested that PMsat may have played some biological role which led to its maintenance in *Peromyscus*^41^.

Another interesting finding of our study is that SATCHO2 is composed by ~ 2292 bp monomers, an uncommonly large size for a satDNA sequence. Most satDNAs identified in plants and animals showed monomer lengths around 150–180 bp and 300–360 bp, respectively^42,43^. There is a limited number of species in which satDNAs with monomers ranging from 1 kb to ~ 2 kb have been reported. That is the case of some whales^44^, South American monkeys^45^, banana^46^, non-domestic Bovidae^47^, and the field bean^48^. SatDNA monomers larger than 2 kb have been identified in bovines^49^ and in the ant *Monomorium subopacum*^50^. To our knowledge, the only examples of monomers significantly larger than SATCHO2 were reported in Bovidae: the satDNA 1.709 (*SATIV*) with ~ 3.8 kb and the satDNA 1.711b with ~ 2.6 kb^49,51^.

Finally, several studies have demonstrated that satDNAs, especially those found in centromeres, are associated with Robertsonian translocations, the main chromosome rearrangements related to Bovidae genome evolution^5,52–55^. It would be interesting to investigate if there is also a link between satDNAs and chromosome rearrangements in Xenarthra, as the number of available genomes of this group will certainly increase in the near future.

## Materials and methods

### Identification and analysis of satDNA sequences

In order to identify the most abundant satDNA sequences in the genomes of *Choloepus* species we performed a graph-based clustering analysis of sequence reads using the pipeline RepeatExplorer2^14^. The analysis was performed in a set of 357,044 random sampled reads (~ 1.19% genome coverage) from the *C. didactylus* genome (accession: SRX4501348) and 789,160 random sampled reads (~ 2.6% genome coverage) from the *C. hoffmanni* genome (accession: SRX282195). Identified satDNA consensus sequences were used as queries in searches conducted on Repbase^56^ and GenBank (https://www.ncbi.nlm.nih.gov/genbank/) in order to detect similarities with previously described sequences. To analyze the satDNA copies directly in the species genomes, we retrieved a sample of each satDNA sequences from the *C. didactylus* (accession: PVKG000000000.1) and *C. hoffmanni* (accession: ABVD00000000.2) assembled genomes available on GenBank using Blastn searches with default parameters^57^. The software DnaSP 6.12.03^58^ was used to identify DNA polymorphisms and nucleotide diversity along the satDNA sequences, by applying a window size of 10 bp (SATCHO1 and SATCHO2) and a step size of 2 bp for SATCHO1 and 3 bp for SATCHO2. Windows that exhibited standard deviation (S.D.) values ≥ 2, from the average variability, were considered highly variable, while those with values ≤ 2 S.D. were considered conserved.

We searched putative CENP-B box-like motifs (CTTCGTTGGAAACGGGA)^36^ on the SATCHO1 and SATCHO2 monomer sequences using the alignment algorithm MUSCLE^59^ on MEGA7^60^. We also searched for dyad symmetries in the satDNA sequences using the EMBOSS palindrome software^61^ with a minimum palindrome length of 4 bp and maximum gap between elements of 20 bp. We used the RNAfold web server (https://rna.tbi.univie.ac.at/)^62^ to search for optimal secondary structure with minimum free energy on the SATCHO1 and SATCHO2 sequences.

Pairwise evolutionary distances within each satDNA sequence from *C. didactylus* and *C. hoffmanni* were estimated using MEGA7^60^. The values were used to obtain non-metric multidimensional scaling (NMDS) ordinations with the R package Vegan^63^, representing Euclidian distances in four dimensions. We used Rstudio v1.1.442^64^ to conduct the NMDS analysis and plotting of the results. We constructed a phylogeny of the sequences using the Neighbor-Joining method with 1000 replicates on MEGA7^60^. The phylogenetic tree was edited using iTOL4.4.1 (https://itol.embl.de/)^65^.

### Biological samples

Chromosome preparations and genomic DNAs were obtained from cultured fibroblasts of *C. hoffmanni* and *M. tridactyla* male individuals. Tissue and blood samples from both specimens were obtained from Fundação de Parques Municipais e Zoobotânica de Belo Horizonte/MG, Brazil, under a license from IBAMA (Instituto Brasileiro do Meio Ambiente e dos Recursos Naturais Renováveis) conceded to M. Svartman (Process Sisbio 28422-5). The *C. hoffmanni* individual came from an unknown location in Rondônia estate, Brazil, and the *M*. *tridactyla* individual was apprehended by IBAMA in Esmeraldas, Minas Gerais, Brazil, but its origin is unknown. We also used the genomic DNA from a male *B. variegatus* captured in Teófilo Otoni, Minas Gerais, Brazil.

Cell cultures and chromosome spreads were obtained according to Stanyon and Galleni^66^ and genomic DNAs were obtained with the Wizard Genomic Purification kit (Promega).

### Molecular analysis

The identified satDNAs were amplified by polymerase chain reaction (PCR) from *C. hoffmanni* genomic DNA with the following primers designed from the consensus sequences generated on RepeatExplorer: SATCHO1-F (AGTTGTTTTTCAGCCCAGGG) and SATCHO1-R (CACGTGGGACTCTGCGAAAG); SATCHO2-F (TCTCACCCGGATCTGAACCT) and SATCHO2-R (GGATACGGGGGTTTGAAGCA). The thermocycling conditions were as follows: 95 °C-5 min, 30 cycles: 95 °C-1 min; 53.4 °C-1 min; 72 °C-1 min; final elongation: 72 °C-10 min. The PCR products were extracted from a 1% agarose gel, purified with Wizard SV Gel and PCR Clean-up System kit (Promega), and cloned into a plasmid vector pGEM-T-Easy cloning kit (Promega). The recombinant plasmids were sequenced with the ABI 3730 platform (Applied Biosystems). The sequences obtained have GenBank accession numbers: MT505303–MT505310.

### Banding patterns and fluorescence in situ hybridization (FISH)

The GTG- and CBG-banding of *C*. *hoffmanni* chromosomes were performed according to Seabright^67^ and Sumner^68^, respectively. FISH was performed using the cloned satDNA sequences as probes after they were labeled by nick-translation with digoxigenin-11-dUTP (DIG-Nick Translation mix, Roche Applied Science) for SATCHO1 and biotin-16-dUTP (Biotin-Nick Translation mix, Roche Applied Science) for SATCHO2. The probes (~ 150 ng in 50% formamide/2xSSC) were denatured for 10 min at 98 °C. Chromosomes were dehydrated in ethanol series (70%, 90%, 100%) and denatured in 70% formamide/2xSSC for 2 min at 75 °C. The hybridization was performed overnight at 37 °C. Post-hybridization washes comprised two 5 min incubations in 2xSSC at 45 °C. Immunodetection was performed with anti-digoxigenin conjugated with rhodamine (Roche Applied Science) for SATCHO1 and avidin conjugated with FITC (Roche Applied Science) for SATCHO2. Chromosomes were counterstained with DAPI 1:500 in Slowfade (Invitrogen). The analysis was performed under a Zeiss Axioimager 2 epifluorescence microscope adapted with a CCD camera and image acquisition was performed with the AxioVision (Zeiss) software (Carl Zeiss MicroImaging, Jena, Germany).

### Verification of SATCHO1 and SATCHO2 in other Xenarthra species

To verify the presence of the identified satDNAs in other Xenarthra species, we conducted Blastn searches on all assembled Xenarthra genomes except *Choloepus*, using SATCHO1 and SATCHO2 consensus sequences as queries. We also performed PCRs with genomic DNAs from *B. variegatus* and *M. tridactyla* using the same set of primers and conditions applied to amplify SATCHO1 and SATCHO2 in *C. hoffmanni*. The genomic DNA of *C. hoffmanni* was used as a positive control. PCR products from *B. variegatus* and *M. tridactyla* were cloned, sequenced (accession numbers: MT505305–MT505308), and used as probes for FISH under the same conditions described above.

## Supplementary information

Supplementary Information.

## Data Availability

The datasets generated during and/or analyzed in the current study are available in the GenBank repository (https://www.ncbi.nlm.nih.gov/genbank/) under accession numbers: MT505303–MT505310.
