# Supplementary material for: Identification and characterization of satellite DNAs in two-toed sloths of the genus Choloepus (Megalonychidae, Xenarthra)
Source: Sci Rep. 2020 Nov 5;10:19202. doi: 10.1038/s41598-020-76199-8 (PMC7644632; doi:10.1038/s41598-020-76199-8)
Supplement: Supplementary file 1 — Supplementary Information. [file 41598_2020_76199_MOESM1_ESM.pdf]

Supplementary Information for

Identification and characterization of satellite DNAs in two-toed sloths of the genus *Choloepus* (Megalonychidae, Xenarthra)

Radarane Santos Sena[1]; Pedro Heringer[1]; Mirela Pelizaro Valeri[1]; Valéria do Socorro Pereira[2]; Gustavo C.S. Kuhn[1]; Marta Svartman[1]*

[1]Laboratório de Citogenômica Evolutiva, Departamento de Genética, Ecologia e Evolução, Instituto de Ciências Biológicas, Universidade Federal de Minas Gerais, Belo Horizonte, MG, Brazil

[2]Fundação de Parques Municipais e Zoobotânica, Belo Horizonte, MG, Brazil

Running Title: Satellite DNAs of two-toed sloths

*Corresponding author
E-mail: svartmanm@ufmg.br
Telephone number: 5531-34092612
Fax number: 5531-34092568

Supplementary Data1:

>SATCHO1

GAAAGCAGTATGTTACACAGTTCTGAAAACTAAGTTCAATACAGCTTTCATTGACTTCCATGTG
TCTTCCCTGGGCTGAAAAACAACTTTTTTGAAAGCAGTCACGTGGGACTCTGC

Supplementary Data2:

>SATCHO2

TGGCCTGTGCAGTCAGGAGTAGGCATATGAGGATGATTCTGCAGCCCTGAGGATGACATGCCCC
CGGGTGGTTGGTGAGATTTTTGACTCTCCCGTTAGTGCGCTGCACTGGTATGCCCGAGGGCAAC
TTCCAGTGGGTTGTGGGCTAGGCCGAGGCTTAGTGAAGTGCCCCTGCCCTCGCTTGGGCCTGCC
ATTCTTTTCCAATGGCACTGATATTACCAGTGGATCGTAGACATGCAGCCTAGCACCCCGAATC
TCACTTAGGAGAGCTCACCGTGAGGCATGCCCATGTTCAGTCAATGAACACTCTAGAAGTCAGG
GAAATTGGGACAACTGTGACGTGTCTGTCCTCTCCACTGTTTATACCCGGCATGATGATTGGTT
CATGGCTCCCTTAATCGTGGGATTTCTTGCTCTATTGGTGCTTGCCGGATCTCTCCTTCTGGGA
CTAAAGGCAGAGTGATAACCAAGGTGGTCCCGTGATGCAGAGACCACATGACATGTGGACAGGC
TGATGTTTTCTGGGTTGAGATTGCTGCAGGCACGGTTGTGGACAGGGACGGAGACTCCCAGAGG
GCCGGCACGAAGGTGGACACTGAATATTTTTCTTGCTCTTTCCAGTCATTTCCATTGCCATTAG
GGAATTCCCTGTTCTGGGAGCCAACATACTCTGGCTCTGTCATTGAGTGTGATCCACAAGGAAC
TGTAAAGGATCACCCCGAGTCCCCCTCTGACCTGCTGCTGGGCATGGCAGAGATTTACCACAGG
CGTGGCCCCTGAACTGTTCCACATCTGAAAGAAGATTTCAGCAAAGGGAGAGTTCTTCCCCGAA
GAATTTAGTTCCTGAATTTGCTGATGCTCAAGGGAGTATGCCCAAGTGGAACTGACTGTGACAA
TTCTTCTCCTATCCCATGAAAGCAGGTCAGTGTAATCTCACCCGGATCTGAACCTAGGCCTTCA
TAAGGATGGAAGCCAACACTTAGGAAGTGTGTGTTTATATAATTCATTTCTTCACCGTGTTAAC
ATTGTTTCACATTCCTTGTATTAAAAGGTTGCCTTTAAGAACTGGACATTTGTCAGTAAAAGAA
TGTCAATTTCTTTAGTTATTCTTGTTGTATTTCCCTTCACAACAGAGACGTTCTCTTAGGTATT
CACTGGAACATTACCACAATCTTTAAATTTACCTGGTCGTAGTACAAATATGTCGCCTAGGGAC
CTGGTCCACACCTCAACAATTAGCCACTCATGACCTATAGACCAAGCAAAATGTTAATGGCAGT
AATAATGTGAGAAATATAGAACGGAAAAAACAAGACAGAGTGTTAGGTCTCACGTGAAACATTC
CGTTTTCTTCAGGTGTATTTATTCTCTTTCCCGTGAATATACATCAATTATTTCCTTCACCTGT
ATAAATTAGAAAACTGCTGAACCATCTTTAAAAACTATTCCCCAAATTCTGTTTTATTCAGAGA
TTCTTCCTGCTGATATTGACTTTATGAGCTTGAGGTGAGATAGGCCGATAATTATTTTGCCCGC
ACTGCGGTTATTTTTTAAATGAAAAGTTTAAATTTTGTTTCTTTCAGTCTATTGAATTCATTAT
ACATATTCTCTGTAAGCTACAAAAACACACAAACATCAAAAAGTCAAAACTTTTCATTAAAGCA
AAGCAGAGTTTAAGAAAAGCAAATAACCTATAGTAACTACTTTGCTTCCTCATTGCTCAAGTTT
GCATTCATGTGTTATCTTCAAGGCATTTGTTTAAAGATTTCTAATATTATAAACAATACTCGTC
CTACAGTCCATTTAGTTCCTTTTGGGTACTTTTTCTTGCTGCACAGACCTGTTTCCCTTTTCCG
TCTCTTGTCATCTAGTTACAGTAACACTGCTTCAAACCCCCGTATCCAGTTTCAGATAGAGGCT
TTTCTGCTTTGCCTAGTGTGCAGACTCAGCTAAAGAGCAGGGAATGTGTTACTGAATTTTTCCC
CTTACCCCACGGAGAAGTCGAAGTGCCCACGTGTGCCCAATAGGCGGCAGGAAATCCCAAGTTA
ACAAAAGTCAGTGAAAAATTCCCACTGGGGCTCGAACCGGTGGAGGAGACAGCGTCAGCACAGG
CTCCAAAGCGGTTTAGAATTCTAGCAGGGGCTCGGAATGCGCATGCGCAAACGACGCTGTACCT
CATCTGTGTCCCAAAGGAATCTGGTTCCTAAGCAACAGACCTCTAGCCCAACGGCTGCCCTGTG
GTCTGAACATGTAAGGGGCTCCCCAAGTGTGCAGGTCACGGTGGAGGTGTTG

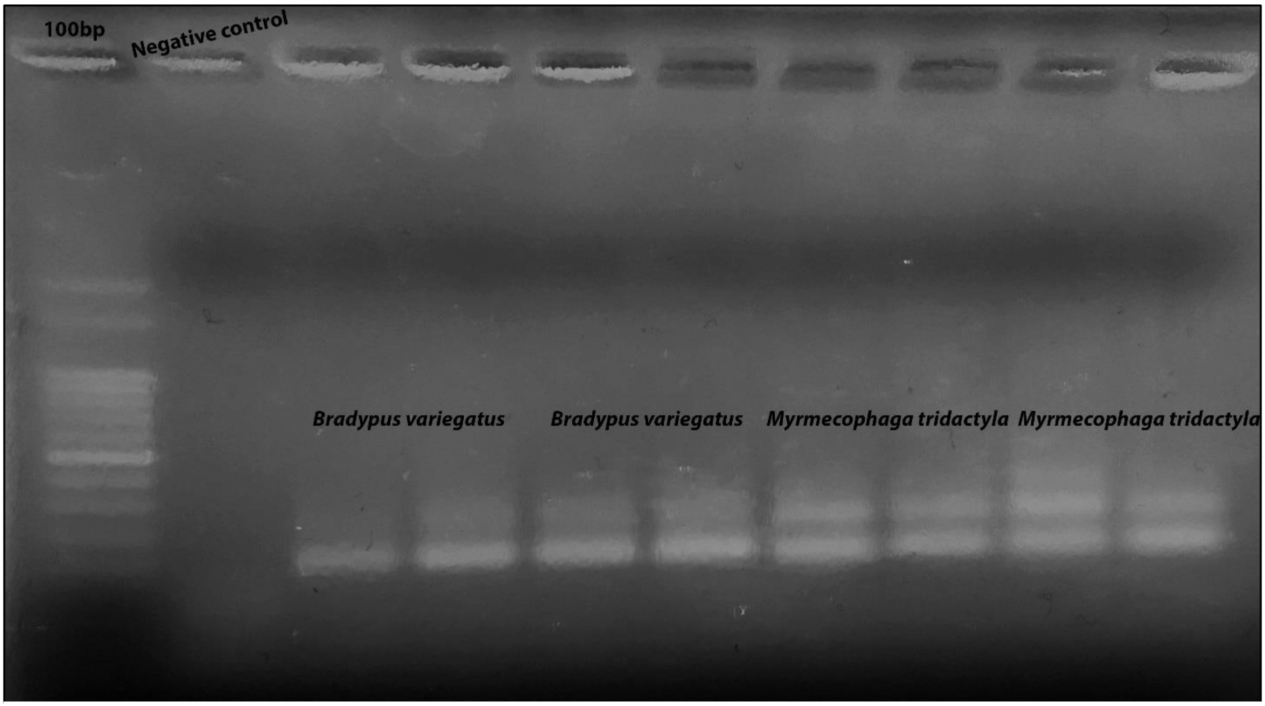

Supplementary Figure 1. Uncropped agarose gel of PCR products from SATCHO1 amplified on genomic DNAs of *Bradypus variegatus* and *Myrmecophaga tridactyla*.

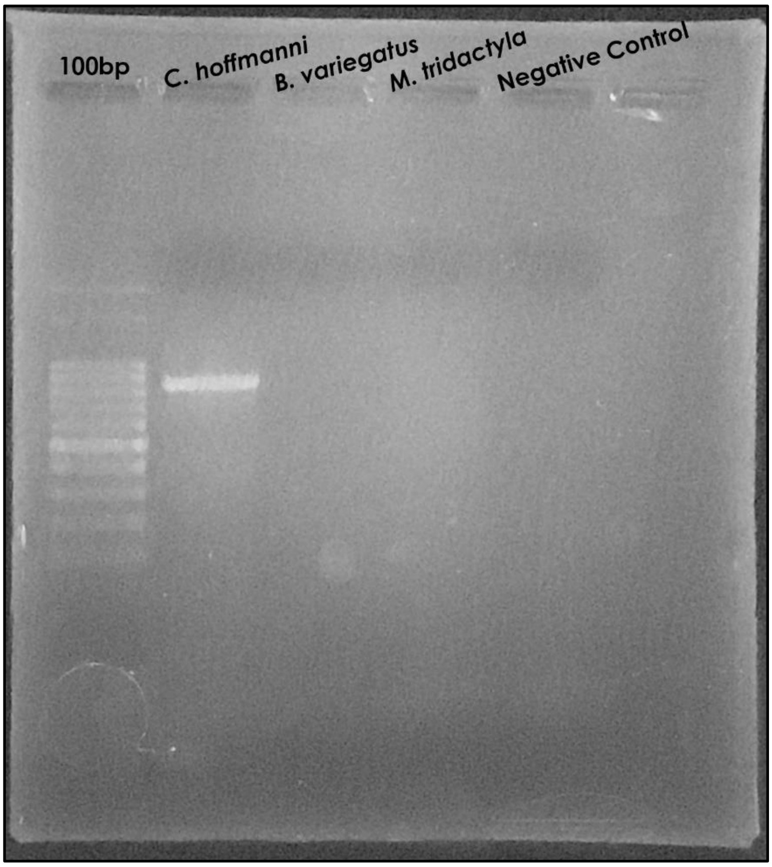

Supplementary Figure 2. Uncropped agarose gel of PCR products from SATCHO2 amplified on genomic DNAs of *Choloepus hoffmanni*, *Bradypus variegatus* and *Myrmecophaga tridactyla*.
